# Effectiveness of Pulmonary Rehabilitation among COVID-19 Patients: A Systematic Review and Meta-Analysis

**DOI:** 10.3390/healthcare10112130

**Published:** 2022-10-26

**Authors:** Sameer Badri AL-Mhanna, Mahaneem Mohamed, Norhayati Mohd Noor, Hafeez Abiola Afolabi, Ahmad Adebayo Irekeola, Kizito Eneye Bello, Monira I. Aldhahi, Wan Syaheedah Wan Ghazali

**Affiliations:** 1 Department of Physiology, School of Medical Sciences, University Sains Malaysia, Kubang Kerian 16150, Kelantan, Malaysia; 2 Department of Family Medicine, School of Medical Sciences, Kubang Kerian 16150, Malaysia; 3 Department of General Surgery, School of Medical Sciences, Hospital University Sains Malaysia HUSM, University Sains Malaysia USM, Kubang Kerian 16150, Kelantan, Malaysia; 4 Department of Medical Microbiology and Parasitology, School of Medical Sciences, University Sains Malaysia, Kubang Kerian 16150, Kelantan, Malaysia; 5 Microbiology Unit, Department of Biological Sciences, College of Natural and Applied Sciences, Summit University Offa, Offa 4412, Nigeria; 6 Department of Microbiology, Kogi State University (Prince Abubakar Audu University), Anyigba 272102, Nigeria; 7 Department of Medical Microbiology and Parasitology, University Sains Malaysia, Kubang Kerian 16150, Kelantan, Malaysia; 8 Department of Rehabilitation Sciences, College of Health and Rehabilitation Sciences, Princess Nourah bint Abdulrahman University, P.O. Box 84428, Riyadh 11671, Saudi Arabia

**Keywords:** SARS-COVID-19, coronal virus disease, long COVID, exercise, physical activity

## Abstract

Background: Many COVID-19 patients presented with detrimental features, such as impaired respiratory function, physical capacity, and overall poor quality of life. The present study evaluates the effectiveness of pulmonary rehabilitation on COVID-19 patients. Methods: We searched PubMed, Scopus, ScienceDirect, and Google Scholar from 2019 to 2021. The protocol was registered in PROSPERO with the registration number CRD42021273618. We performed statistical analyses via random effects and expressed the outcomes as standardized mean difference (SMD) for continuous variables, with 95% confidence intervals (CI). Results: We included six trials involving 432 patients. The primary outcome showed a significant improvement in physical function (SMD 0.83, 95% CI −0.58 to 1.09; *p* < 0.001; four trials, 266 participants; high-quality evidence). There was significant difference in anxiety (SMD −0.80, 95% CI −1.23 to −0.37; *p* = 0.003), physical activity intensity levels (SMD −1.27, 95% CI −2.23 to −0.32; *p* = 0.009), sleep quality (MD −0.05, 95% CI −0.83 to −0.16; *p* = 0.004), peripheral muscle performance of lower limbs (SMD 0.90, 95% CI −0.60 to 1.20; *p* < 0.001), and dyspnoea outcomes (SMD −0.55, 95% CI −0.87 to −0.23; *p* = 0.007). Conclusions: Pulmonary rehabilitation is an effective adjuvant therapy that minimizes COVID-19 severity in the intervention group compared to the conventional treatment. The findings of this study will need to be considered in the framework of the clinical outcome as observed in the intervention outcome. Additionally, safer data on guideline rehabilitation would be needed to examine whether pulmonary rehabilitation would be a fruitful intervention to reduce COVID-19 severity.

## 1. Introduction

In China’s Hubei region, an abrupt occurrence of a contagious respiratory disease called COVID-19 was announced by the WHO in 2019 and spread universally [1]. By 15 October 2019, 38,394,169 confirmed cases and 1,089,047 mortalities had been reported by the WHO worldwide [2]. It was declared a pandemic as it spread over 170 nations and over 30 million people worldwide, causing the deaths of over 1 million people as of 17 September 2020. COVID-19 has created a devastating blow to the world’s population, culminating in over 2.9 million fatalities worldwide, designating it as the most serious world health crisis since the 1918 influenza pandemic [3]. Furthermore, COVID-19 infections have been found in 235 nations, regions, or territories worldwide [4].

Patients experience COVID-related symptoms, and the severity of the condition might escalate to respiratory distress or respiratory failure, necessitating the use of an intensive care unit [5,6]. Many COVID-19 patients have chronic clinical manifestations at the beginning of the disease and after discharge from acute treatment, including impaired respiratory function, decreased physical activity, and a worse quality of life [7,8]. Hao, Tan [9] reported that infectious disease outbreaks, such as COVID-19, are linked to psychological stress, as well as signs of mental illnesses, including a higher incidence of anxiety, sadness, distress, and depression, which are known to influence the quality of life [10]. Furthermore, social remoteness—doing work and studying at home, having dependent kids, and having less physical touch with others—is a substantial source of psychosocial stressors [11]. Moreover, COVID-19 might have detrimental sequelae even after the post-acute phase, depicting a new pathological condition [12].

The COVID-19 pandemic has created a rising public fear that can lead to non-healthy lifestyle changes such as inadequate eating habits and lack of physical activities [13]. Moreover, the provision of conventional inpatient or outpatient rehabilitation is compounded through decreased capacity in post-acute care as well as medical and public health actions enacted to lessen the risk of virus transmission. Rehabilitation may improve symptoms and quality of life following COVID-19 infection [14]. Respiratory function, psychological wellbeing, dyspnoea, and physical function, such as peripheral muscle function, have all been reported to be impaired in patients with COVID-19, particularly in the elderly [15,16]. With the observation of the improved condition of the discharged COVID-19 patients, pulmonary rehabilitation will enhance the prognosis and functional status and improve the value of life, but investigations on the result of this intervention are lacking worldwide [17]. The feasibility and prospective benefit of pulmonary rehabilitation in COVID-19 patients remain unclear [18]. 

However, so far, there is a lack of data from highly rated trials on the efficacy of pulmonary rehabilitation in COVID-19 patients [19]. Therefore, we have conducted a systematic review and meta-analysis to assess the effectiveness of pulmonary rehabilitation on physical function, quality of life, and respiratory-related symptoms and psychological factors among COVID-19 patients.

## 2. Materials and Methods

### 2.1. Protocol and Registration

The protocol was registered in PROSPERO with the registration number CRD42021273618.

### 2.2. Research Question

Studies about the effectiveness of respiratory rehabilitation among COVID-19 patients were selected based on the “PICOS” (PRISMA-P 2016) technique:

- “PICOS”
- P (Population) = COVID-19 patients
- I (Intervention) = respiratory rehabilitation
- C (Comparison) = standard treatment
- O (Outcome) = physical function and quality of life
- S (Study design) = randomized controlled trial, and controlled clinical studies.

### 2.3. Types of Outcome Measures

#### 2.3.1. Primary Outcomes

1. Physical function.
2. Quality of life.

#### 2.3.2. Secondary Outcomes

3. Dyspnoea.
4. Respiratory function.
5. Physical activity intensity level.
6. Anxiety.
7. Depression.
8. Peripheral muscle performance of lower limbs.

### 2.4. Data Sources

Two independent authors (S.B.A. and H.A.) conducted an electronic literature search up to September 1 by combining MeSH terminology and keywords with the Boolean operators “OR” and “AND” to find relevant literature. The keywords are (“physical activit*” OR “exercise” OR “pulmonary rehabilitation” OR “telerehabilitation” OR “Respiratory rehabilitation” OR “training” OR “fitness”) AND (“Covid-19” OR “SARS-CoV-2” OR “2019-nCoV”) (Supplementary File S1).

### 2.5. Eligibility Criteria

A literature search was carried out to identify experiments that investigated the impact of exercise on COVID-19 patients published between 2019 and 2021. Two authors (S.B.A. and H.A.) used the PICOS strategy to examine the extensive texts of the remaining papers and define the inclusion and exclusion criteria. The judgment of a third reviewer (A.A.I.) was employed to settle disagreements. 

#### 2.5.1. Inclusion Criteria

9. Unvaccinated COVID-19 patients with no age limit.
10. Publications with no language limitation and with full text available.
11. Pulmonary rehabilitation.
12. Randomized controlled trials, and controlled clinical studies.

#### 2.5.2. Exclusion Criteria 

All review articles, case reports, commentary, letters, and short communication.

### 2.6. Study Selection

Two authors, S.B.A. and H.A. scanned the papers based on a linear evaluation of names, abstracts, and complete texts (in cases of doubt). The remaining articles were evaluated entirely based on the qualifying criteria before making a final selection. This method was used independently, with the assistance of a third researcher (A.A.I.) in the case of any conflicts or doubts.

### 2.7. Data Extraction

After reading the full article, two authors (S.B.A. and A.A.I.) conducted independent sampling and data extraction from qualifying studies. The studies that were included produced substantial data, which was extracted and published. This includes the first author, journal name, population, year of publication, gender, method (exercise name, duration, intensity, sets, reps, intervention timing, study duration, and outcome measures. 

### 2.8. Assessment of Risk of Bias

We checked the risk of bias based on random sequence generation, allocation concealment, blinding of participants and personnel, blinding of outcome assessors, completeness of outcome data, selectivity of outcome reporting, and other bias, as discussed in the Cochrane Handbook for Systematic Reviews of Interventions [20].

### 2.9. Analysis

#### 2.9.1. Measurement of Treatment Effect

We used risk ratios (RR) and 95% confidence interval (CI) to draw forest plots for trials with categorical variables, and estimation of risk differences (RD) and 95% CI were reported as well. If the outcomes were continuous variables, we planned to analyse the data using mean differences (MD) or standardized mean difference (SMD) and 95% CI. We determined the presence of heterogeneity in two phases. First, we compared demographics, contexts, treatments, and outcomes to see any noticeable variability. Second, we used the I^2^ statistic [21] to analyse statistical heterogeneity. We conducted a subgroup evaluation on the duration of intervention when it was feasible.

#### 2.9.2. Sensitivity Analysis

We ran a sensitivity analysis to see how the risk of bias affected sequence generation and allocation concealment in the papers that were included.

#### 2.9.3. Summary of Findings Table

We used the Cochrane Collaboration’s Grades of Recommendation, Assessment, Development, and Evaluation (GRADE) approach to evaluate the quality of evidence in systematic reviews. The GRADE system defines four levels of quality, with randomized trial evidence being the highest. Depending on the existence of four elements, it might be degraded as moderate, low, or even extremely poor-quality evidence: (i) constraints in study design and implementation; (ii) indirectness of evidence; (iii) unexplained heterogeneity or inconsistency of results; and (iv) imprecision of outcomes. The GRADEpro software was used to show the quality of evidence for each particular outcome, and the evaluation is being phased in alongside the ‘Summary of findings’ (SoF) table [21].

The SoF table is made up of the following elements:

- Key findings that were summarized (participants, comparative and baseline data, and results) [22].
- Statistical results that have been condensed.
- A summary of the evidence’s quality, the degree of the effect, and the source of information utilized in the assumed risk.

## 3. Results

**Figure 1:**
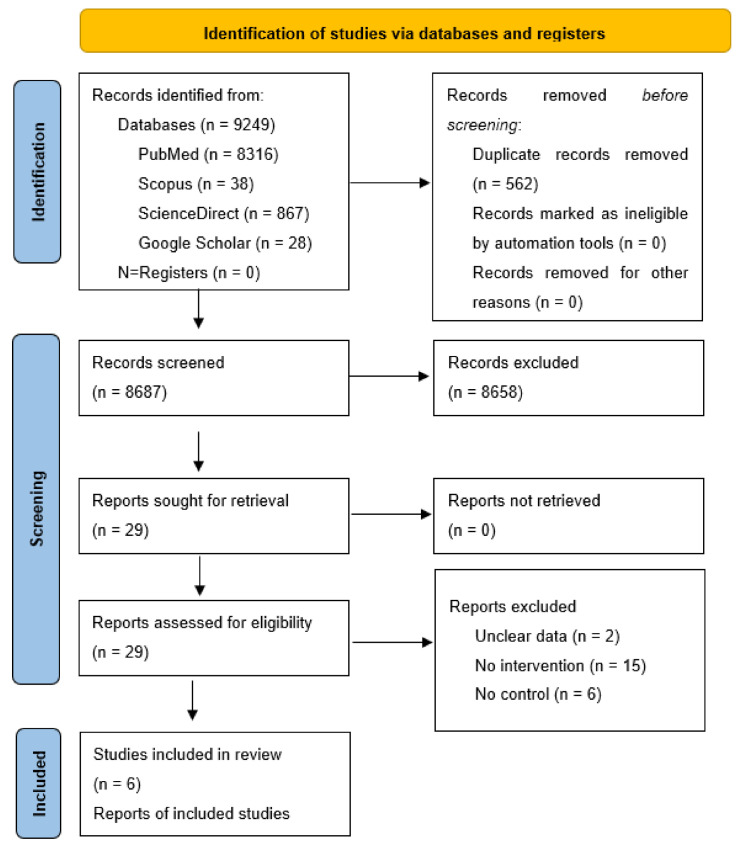
PRISMA flowchart for search strategy.

A total of 9249 studies were retrieved from PubMed, Scopus, Science Direct, and Google scholar using all MeSH keywords in Figure 1. After identifying duplicate articles, a total of 8687 studies were screened for further selection. After reading the articles’ title and abstract, a total of 8605 were excluded according to our inclusion and exclusion criteria. Thus, the remaining 82 articles were proceeded for further selection by reading the full texts, out of which 76 were excluded. The remaining 6 articles that met the eligibility criteria were used for data extraction.

### 3.1. Included Studies

We included six trials with a total of 432 participants [17,23,24,25,26,27]. All six trials contributed to the primary outcome. 

### 3.2. Participants

Two out of the six trials were from high-income countries [23,24], and four trials were from middle-income countries [17,25,26,27]. Four out of the six trials recruited their respondents from hospital settings [17,25,26,27], while two trials reported enrolling their participants through an informative text or email distributed on social media platforms (WhatsApp and Facebook), television stations, and newspapers, all of which included interviews with members of the research team [23,24]. Three of the six trials performed their exercise at the hospital [13,22,23], while three conducted the exercise at their home [23,24,25]. Table 1 describes the characteristics of the included trials.

Two trials excluded patients if the patient had any of the following diseases: chronic kidney disease, respiratory conditions in the last 12 months, chronic neurological disease, chronic lung disease, and hypertension [23,24]. One trial reported excluding patients if they did not have a smartphone and had cognitive dysfunction [26]. One trial included only those with mild COVID-19 infection without detailing the exclusion criteria [27]. One trial excluded patients with moderate or severe heart disease with haemorrhagic stroke or neurodegenerative diseases [17]. One trial excluded patients from the study if they had dyspnoea of 4–5 episodes, a resting heart rate of more than 100 bpm, uncontrolled chronic illness (e.g., diabetes mellitus with random blood glucose > 16.7 mmol/L, haemoglobin hbA1C > 7.0%), cerebrovascular disease, mental disorder, and participated in other rehabilitation programmes [25].

Four trials reported more female recruitment rates than males: overall percentages for females ranged from 51% to 59% [24,25,26,27]. Two trials reported more males than females: overall percentages for males ranged from 55% to 68% [17,23]. 

On comorbidity, in one trial, 50 out of 72 COVID-19 patients reported having hypertension, type 2 diabetes, and osteoporosis [17]. Out of 120 COVID-19 survivors, 45 patients reported single comorbidity, while multi-comorbidities such as heart disease, hypertension, diabetes, lung disease, and obesity were reported in 28 COVID-19 survivors [25]. 

### 3.3. Intervention

Patients in our included studies were randomly assigned to intervention and control groups. In two trials, the intervention was a pulmonary telerehabilitation programme [23,25]. One trial combined a telerehabilitation programme with additional exercises [24]. In two trials, the intervention was a pulmonary rehabilitation programme [17,27]. In one trial, the intervention was a breathing exercise [26]. 

There was a difference in the duration of the intervention between our included studies. In three trials, the intervention was one week [23,24,27]. In another trial, the intervention was for 10 days [26]. In two trials, the intervention was for six weeks [17,25]. In three trials, the intervention was done at home [23,24,25], while another three trials were conducted in the hospital [17,26,27].

### 3.4. Comparison

We compared COVID-19 patients who underwent pulmonary/respiratory rehabilitation or telerehabilitation to the control group who received only standard treatment [17,23,24,25,26,27].

### 3.5. Excluded Studies

Out of 29 full-text articles, 23 were exempted because they did not satisfy our inclusion criteria, including 1 trial that did not report on the effectiveness of pulmonary/respiratory rehabilitation among COVID-19 patients [28], 2 studies with unclear data, 15 articles with no intervention, and 6 with no control. 

### 3.6. Risk of Bias in Included Studies

**Figure 2:**
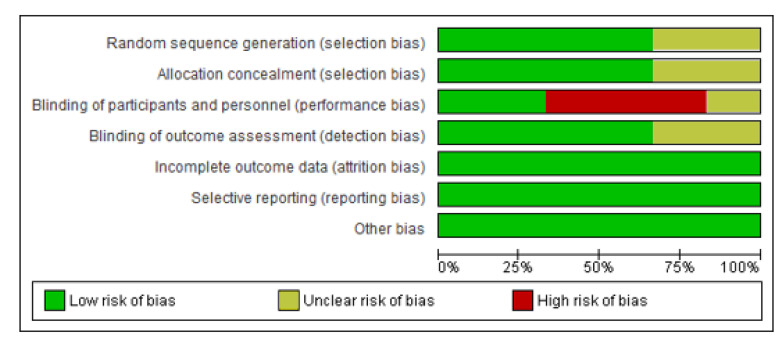
Risk of bias graph: review authors’ judgements about each risk of bias item presented as percentages across all included studies.

**Figure 3:**
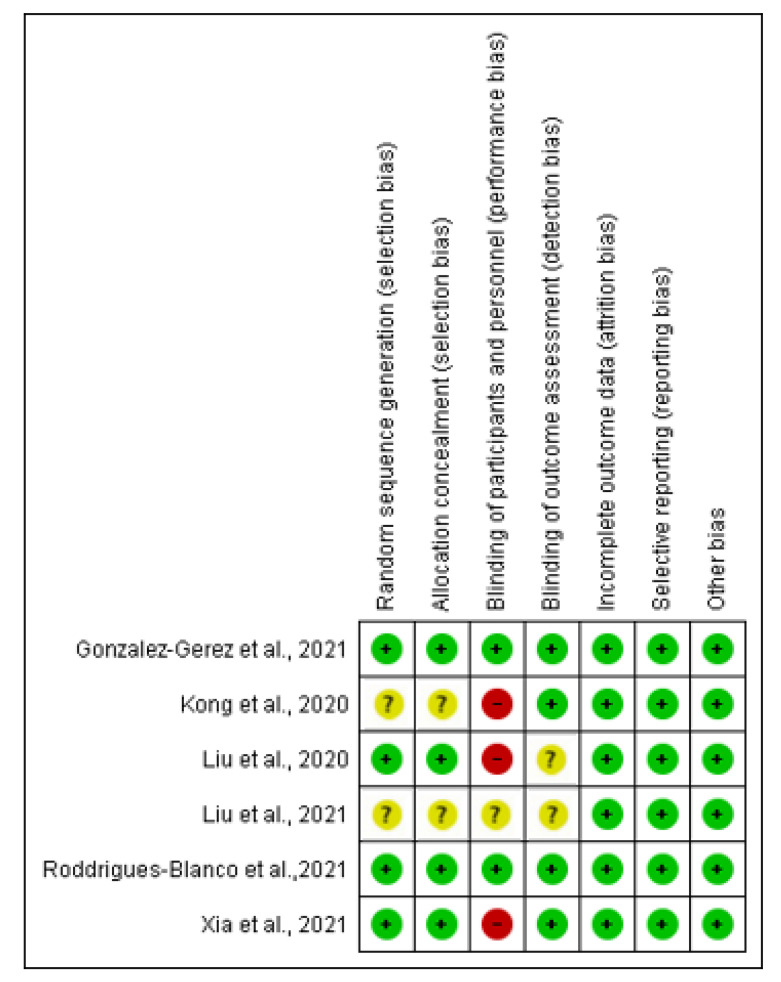
Risk of bias summary: review authors’ judgements about each risk of bias item for each included study [17,23,24,25,26,27].

Figure 2 and Figure 3 show the risk of bias result assessment. For each risk of bias indicator, Figure 2 shows the proportion of studies classified as a low or unclear risk of bias. The risk of bias indicators for individual studies are shown in Figure 3. The details of these trials are found in the table of ‘Characteristics of the included studies’.

#### 3.6.1. Random Sequence and Allocation Concealment

The method of randomization was described in four trials and the random sequence generation was judged as a low risk of bias [17,23,24,25].

In one trial, the allocation was concealed by central randomization [25]. Liu, Zhang [17] applied computer-generated randomization, and two trials used balanced randomization [23,24]. In the remaining two trials, the method of randomization was not described; thus, we judged random sequence generation as an unclear risk of bias [26,27] (Supplementary File S1).

#### 3.6.2. Blinding of Participants, Personnel, and Outcome Assessment

In two trials, the patients were blinded during the entire study process and based on the blinding of participants, and the trials were deemed to have a low risk of bias [23,24]. In one trial, one patient in the control group was randomized mistakenly, and the blinding of participants was, thus, judged as a high risk of bias [25]. In one trial, the blinding of the patients was not feasible; thus, the blinding of participants was evaluated as a high risk of bias [26]. In one trial, the patients were aware of all rehabilitation procedures; thus, the blinding of participants was judged as a high risk of bias [17]. The information regarding the blinding of the participant was not provided in one trial and was therefore judged an unclear risk of bias [27]. The assessors were blinded in four trials, and blinding of the outcome assessment was judged a low risk of bias [23,24,25,26]. Meanwhile, two trials did not report if the assessors were blinded and blinding of the outcome were judged an unclear risk of bias [17,27]; however, Liu, Zhang [17] stated that efforts had been made to blind assessors and participants to group allocation, but this cannot be guaranteed.

#### 3.6.3. Incomplete Outcome Data

Six trials measured the primary outcomes and were included in the meta-analysis. In two trials, the intervention was a respiratory telerehabilitation programme [23,25]. One trial combined a telerehabilitation programme with additional exercises [24]. In two trials, the intervention was a pulmonary rehabilitation programme [17,27]. In one trial, the intervention was a breathing exercise [26]. Five trials reported that all participants completed the study, and the bias due to incomplete outcome data was judged as a low risk [17,23,24,26,27]. Of the five trials, three trials measured the primary outcome at one week [23,24,27], one trial at 10 days [26], and one trial at six weeks [17]. The sixth trial also measured the primary outcome at six weeks [25]; however, six patients of the intervention group (10%) did not complete the post-treatment assessment. Two patients who discontinued the intervention, one because of chest pain and one for unspecified reasons, missed the post-treatment evaluation but returned for the follow-up assessment. Contact was lost with four additional patients in the telerehabilitation programme group and five patients from the control group at the final follow-up. However, intention to treat analysis was applied, and the incomplete outcome data was judged to have a low risk of bias [25].

#### 3.6.4. Selective Reporting

All six trials reported the outcomes as specified in their methods section [17,23,24,25,26,27] and were regarded as low risks of bias.

#### 3.6.5. Other Potential Sources of Bias

We detected no other potential sources of bias.

### 3.7. Outcomes

The primary outcomes in this review were physical function and quality of life. The physical function of participants in the four trials was measured using a six-minute walking test before and after the intervention [17,23,24,25]. Four trials reported on the quality of life using sleep quality score [27], social support scale [17,26], the physical function of short Form Health Survey-12 and Short Form-36 [17,25], and the mental function of Short Form Health Survey-12 and Short Form-36 [17,25].

The secondary outcomes were dyspnoea, pulmonary function, physical activity intensity, anxiety, depression, and peripheral muscle performance of lower limp. Two trials reported on dyspnoea using the Multidimensional Dyspnoea-12 questionnaire [23], and the modified Medical Research Council questionnaire [25]. Two trials reported on pulmonary function post-intervention using a spirometer [17,25]. Two trials reported on physical activity intensity levels post-intervention using the Perceived Exertion Scale [23,24]. Three trials reported on anxiety post-intervention using the anxiety scale [17,26,27]. Two trials reported on depression post-intervention using the self-depression scale [17,26]. Three trials reported on peripheral muscle performance of lower limp post-intervention using a 30 s sit-to-stand test [23,24], while one trial used squat time [25]. 

#### 3.7.1. Primary Outcomes

The primary outcomes in this review were physical function and quality of life. A “six-minute walk test” was used in four trials to assess physical function [17,23,24,25], while “physical health score” and “mental health score” were used in two trials to assess the quality of life [17,25].

##### Physical Function

**Figure 4:**
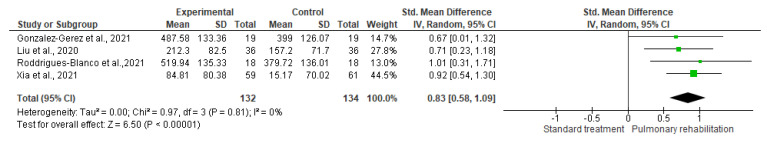
Forest plot analysis of the effect of pulmonary rehabilitation on physical function [17,23,24,25].

Pulmonary rehabilitation improved physical function (SMD 0.83, 95% CI −0.58 to 1.09; I^2^ statistic = 0%; *p* < 0.001; four trials, 266 participants; high-quality evidence) [17,23,24,25] (Figure 4, Table 2) compared to the standard treatment group.

##### Quality of Life

**Figure 5:**
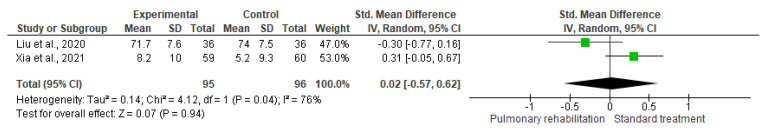
Forest plot analysis of the effect of pulmonary rehabilitation on physical-health-related quality of life [17,25].

**Figure 6:**
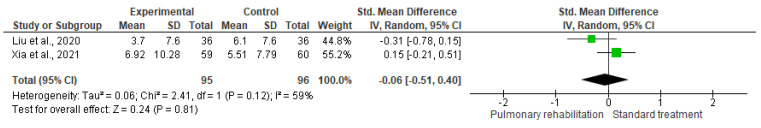
Forest plot analysis of the effect of pulmonary rehabilitation on mental-health-related quality of life [17,25].

There was no significant difference in physical health-related quality of life (SMD 0.02, 95% CI −0.57 to 0.62; I^2^ statistic = 76; *p* = 0.94; two trials, 191 participants; high-quality evidence) [17,25] (Figure 5, Table 2) and mental health-related quality of life (SMD −0.06, 95% CI −0.51 to 0.40; I^2^ statistic = 59%; *p* = 0.81; two trials, 191 participants) [17,25] (Figure 6, Table 2) between the pulmonary rehabilitation group and standard treatment group.

#### 3.7.2. Secondary Outcomes

##### Depression

**Figure 7:**
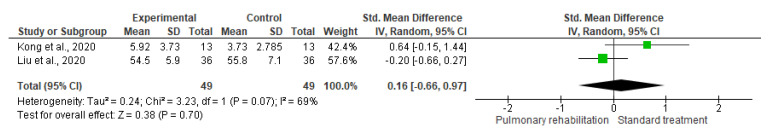
Forest plot analysis of the effect of pulmonary rehabilitation on depression [17,26].

There was no significant difference in depression (SMD 0.16, 95% CI −0.66 to 0.97; I^2^ statistic = 69%; *p* = 0.70; two trials, 98 participants; high-quality evidence) [17,26] (Figure 7, Table 2) between the pulmonary rehabilitation group and standard treatment group.

##### Anxiety

**Figure 8:**
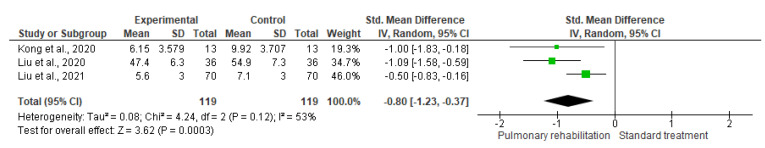
Forest plot analysis of the effect of pulmonary rehabilitation on anxiety [17,26,27].

Pulmonary rehabilitation improved anxiety (SMD −0.80, 95% CI −1.23 to −0.37; I^2^ statistic = 53%; *p* = 0.003; three trials, 238 participants; high-quality evidence) [17,26,27] (Figure 8, Table 2) compared to the standard treatment group.

##### Dyspnoea

**Figure 9:**
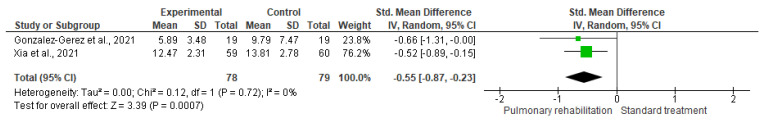
Forest plot analysis of the effect of respiratory rehabilitation on dyspnoea [23,25].

Pulmonary rehabilitation improved dyspnoea (SMD −0.55, 95% CI −0.87 to −0.23; I^2^ statistic = 0%; *p* = 0.07; two trials, 157 participants; high-quality evidence) [23,25] (Figure 9, Table 2) compared to standard treatment group. 

##### Physical Activity Intensity Level

**Figure 10:**
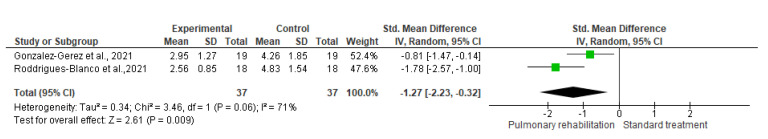
Forest plot analysis of the effect of respiratory rehabilitation on physical activity intensity level [23,24].

Pulmonary rehabilitation improved physical activity intensity level (SMD −1.27, 95% CI −2.23 to −0.32; I^2^ statistic = 71%; *p* = 0.009; two trials, 74 participants; high-quality evidence) [23,24] (Figure 10, Table 2) compared to standard treatment group.

##### Peripheral Muscle Performance of Lower Limbs

**Figure 11:**
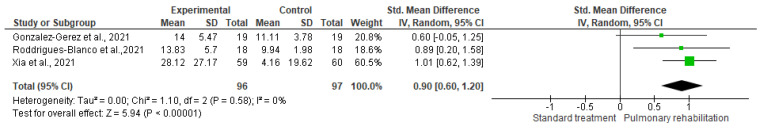
Forest plot analysis of the effect of respiratory rehabilitation on peripheral muscle performance of lower limp [23,24,25].

Pulmonary rehabilitation improved peripheral muscle performance of lower limb (SMD 0.90, 95% CI −0.60 to 1.20; I^2^ statistic = 0%; *p* < 0.001; three trials, 191 participants; high-quality evidence) [23,24,25] (Figure 11, Table 2) compared to standard treatment group.

##### Pulmonary Function

**Figure 12:**
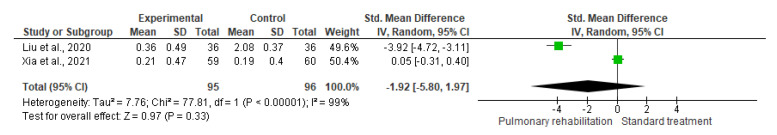
Forest plot analysis of the effect of pulmonary rehabilitation on pulmonary function [17,25].

There was no significant difference in pulmonary function (SMD −1.92, 95% CI −5.80 to 1.97; I^2^ statistic = 99%; *p* = 0.33; two trials, 191 participants; high-quality evidence) [17,25] (Figure 12, Table 2) between the pulmonary rehabilitation group and standard treatment group.

##### Sleep Quality

Pulmonary rehabilitation improved sleep quality (MD −0.05, 95% CI −0.83 to −0.16; I^2^ statistic = 0%; *p* = 0.004; one trial, 70 participants; high-quality evidence) [27] (Table 2) compared to standard treatment group.

##### Social Support

**Figure 13:**
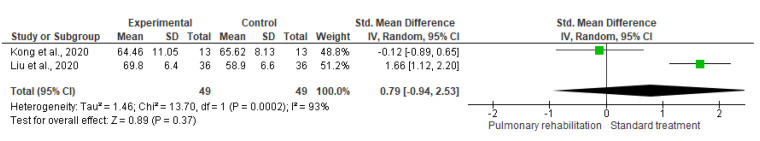
Forest plot analysis of the effect of pulmonary rehabilitation on social support [17,26].

There was no significant difference in social support (SMD 0.79, 95% CI −0.94 to 2.53; I^2^ statistic = 93%; *p* = 0.37; two trials, 98 participants; high-quality evidence) [17,26] (Figure 13, Table 2) between the pulmonary rehabilitation group and standard treatment group.

## 4. Discussion

### 4.1. Summary of Main Results

The present review was designed to incorporate all randomized controlled trials evaluating the effectiveness of pulmonary rehabilitation among COVID-19 patients. There was a significant difference in physical function, anxiety, dyspnoea, physical activity intensity levels, sleep quality, and peripheral muscle performance of the lower limbs in the intervention group following pulmonary rehabilitation compared to the standard treatment group. There was no difference in the quality of life, pulmonary function, depression, and social support outcomes between the intervention and standard treatment groups for the limited number of trials included.

### 4.2. Overall Completeness and Applicability of Evidence

We conducted a detailed and elaborated literature review to evaluate the effectiveness of pulmonary rehabilitation among COVID-19 patients. The RCT included in this review comprehensively illustrate pulmonary rehabilitation outcome among COVID-19 patients. Six trials were included in the meta-analysis. We detected a significant improvement in the intervention groups for the various parameters: physical function, anxiety, dyspnoea, peripheral muscle performance of the lower limbs, physical activity intensity level, and sleep quality. 

### 4.3. Quality of the Evidence

The quality of trial evidence varies from moderate to very low certainty. For most trials in most domains, there was a low or unclear risk of bias. There was no evidence of selective reporting bias. In the original research and subsequent review, a lack of proper random sequence generation may contribute to treatment effect bias in the original trial and the subsequent review. The risk of performance bias was presented in three trials. Performance bias was unclear in one trial due to the lack of information regarding the blinding of the participant. Only one trial reported a loss to follow-up of 10% among the intervention groups as they did not complete the post-treatment assessment, and intention-to-treat analysis was carried out. The random-effect meta-analysis of the study showed low to moderate heterogeneity, where we have done random-effects meta-analysis, there was no shift in the effect estimate, and although the 95% (CI) was wider in all cases, the overall level of evidence contributing to this review as assessed using the GRADE approach is moderate to very low quality. 

### 4.4. Potential Biases in the Review Process

We intended to limit publication bias by exploring several databases without language restrictions and analysing the reference lists of all relevant studies for additional information. All included studies met all the inclusion, and we did not introduce any bias during the review process, all the studies were vividly reviewed and checked for secondary citation. 

### 4.5. Limitation of the Study

All the studies included in this meta-analysis illustrate a similar direction of effect; however, we discovered substantial heterogeneity for quality-of-life outcome. In our analysis, we were unable to explain this due to limited trials. We cannot say with absolute certainty that we have identified all the trials in this field. Considering the fact that there were six trials included, we were unable to create a funnel plot for publication bias relating to each outcome.

### 4.6. Agreements and Disagreements with Other Studies or Reviews

To the best of our knowledge, this represents the first systematic review and meta-analysis been carried out to determine the effectiveness of pulmonary rehabilitation among COVID-19 patients. Three different reviews examined the rehabilitation programme for COVID-19 patients [29,30,31]. Fila, Rocco [29] evaluated the effects of a rehabilitation programme on COVID-19 patients and showed significant improvement in dyspnoea, respiratory function, quality of life, and anxiety among the patients who participated in the rehabilitation programme. The study included 32 articles. Goodwin, Allan [30] included six cohort studies examining longitudinal changes in physical function, three cross-sectional studies investigating the difference in physical function and fitness compared with healthy controls, and one randomized controlled trial investigating the effects of an exercise intervention following SARS-CoV infection. Bernal-Utrera, Anarte-Lazo [31] conducted a scoping review to evaluate the effects of rehabilitation in 29 studies on COVID-19 patients and found a reduction in the severity and progress of COVID-19-related diseases, improved quality of life, and pulmonary function.

## 5. Conclusions

### 5.1. Implications for Practice

Pulmonary rehabilitation has a significant effect on improving physical function, pulmonary function, dyspnoea, anxiety, depression, physical activity intensity level, and sleep quality. Hence, encouraging pulmonary rehabilitation only to minimize COVID-19 infection without adhering to other established standard medical treatment does not seem to be justified, but it might be valuable to patients’ adjuvant therapy. Nevertheless, pulmonary rehabilitation will ensure an effective adjuvant therapy to the conventional treatment, thus improving pulmonary function and quality of life. Though, in communities with a low occurrence of COVID-19 transmission, where a conservative approach is practiced as a means of reducing the disease burden, the impact of this finding would be less appreciated practical-wise. The findings of this review would need to be considered in the context of the clinical outcome as observed in the intervention outcome. Additionally, safer data on guideline rehabilitation would be needed to examine adequately whether pulmonary rehabilitation would be a fruitful intervention to reduce the COVID-19 severity.

### 5.2. Implications for Research

If further studies were conducted to examine the use of pulmonary rehabilitation in COVID-19 patients, they should include a comprehensive physical function test as an outcome and outline safety information. Data on aerobic exercise and resistance exercise for COVID-19 and other respiratory infections should also be collated. Suppose studies are done in remote and less privileged regions or settings with poor access to standard medical care, the adjuvant treatment should include a structured and tolerable pulmonary rehabilitation programme of sufficient duration to ensure the progression of COVID-19 infection and severity are controlled.

## Figures and Tables

**Table 1:** Characteristics of the included trials.

Reference	Journal	Population	Year of Publication	Sample Size (F)	Participants/Age	Period of Recruitment	Gender	Method (Exercise Name, Duration, Intensity, Sets, Reps).	Status of the Patient at Intervention	Duration of Intervention	Outcome Measures	PRO Measure Instrument
1. [17]	Complementary Therapies in Clinical Practice	China	2020	72 EX = 36 CO = 36	Elderly patients with COVID-19. 69.4 ± 8.0	1 January 2020 to 6 February 2020	Male	Participant subjected to pulmonary rehabilitation of two S/W for ten M. The intervention includes respiratory muscle EX, cough EX, diaphragmatic EX: stretching EX at 60% of the individual’s maximal expiratory mouth pressure. While the CO received only standard treatment	Definite diagnosis of COVID-19	Six W	1. PFT 2. functional tests 3. QOL 4. Activities of daily living 5. Mental status tests.	1. Spirometer 2. 6MWT 3. SF-36 4. FIM scores 5. SAS anxiety and SDS depression scores
2. [26]	Frontiers in Psychiatry	China	2020	16 EX = 13 CO = 13	COVID-19 patients 49.98 ± 13.73	23 February 2020 to 13 March 2020	Male and female	Patients undergo breathing exercises for 20 M every D, to stimulate nasal and diaphragmatic breathing, increase expiratory duration, reduce respiratory flow, and regulate breathing rhythm + psychological support The CO received only Standard treatment	Definite diagnosis of COVID-19	10 D	1. Depression and anxiety, 2. Social support	1. HADS-A and HADS-D 2. PSSS
3. [23]	International Journal of Environmental Research and Public Health	Spain	2021	38 Ex = 19 Co = 19	Mild to moderate symptomatology in the acute stage COVID-19 patients 40.79 ± 9.84	19 October 2020	Male and female	Respiratory telerehabilitation programme, the breathing EX was once /D for seven D at home; based on the assessment of the RPE, patients underwent four RPE on a scale of seven-ten for ten M). The CO received only Standard treatment	Confined COVID-19 Patients in the Acute Phase	One W	1. Physical function 2. Peripheral muscle performance of lower limbs 3. Multidimensional nature of dyspnoea 4. Physical activity intensity level	1. 6MWT 2. Thirty-Second STST 3. Multidimensional Dyspnoea-12 4. RPE
4. [27]	Psychology, Health & Medicine	China	2021	140 Ex = 70 Co = 70	patients with mild COVID-19 infections All age group	In March 2020	Male and female	Pulmonary rehabilitation included Five-tone breathing EX, five-step breathing EX and two-section motion E, for seven D. The CO received only Standard treatment	Definite diagnosis of COVID-19	One W	1. Anxiety state 2. Sleep quality	1. SAI 2. PQSI
5. [24]	Medicine	Spain	2021	36 EX = 18, CO = 18	COVID-19 patients with mild to moderate symptomatology 39.39 ± 1174	23 October 2020		Telerehabilitation programme included ten EX based on non-specific toning EX of resistance and strength one/D for seven D using the RPE scale to determine EX intensity. The CO received only Standard treatment	Confined COVID-19 Patients in the Acute Phase	One W	1. Physical function 2. Peripheral muscle performance of lower limbs. 3. Physical activity intensity level	1. 6MWT 2. 30STST 3. RPE
6. [25]	Rehabilitation	China	2021	120 EX = 59 CO = 61	Hospitalized COVID-19 survivors with remaining dyspnoea 49.17 ± 10.75	28 May 2020	Male and female	Participants were subjected to six W of unsupervised home EX programme for three-four S/W. Ex includes: -breathing control and thoracic expansion. The CO received only Standard treatment	In post-discharge COCID-19 patients	six W	1. Physical function, 2. Peripheral muscle performance of lower limp 3. Pulmonary function, 4. QOL 5. Dyspnoea	1. 6MWT 2. squat time in seconds 3. Spirometer 4. HRQOL12 5. mMRC dyspnoea

EX = exercise; CO = control group; RT = resistance training; AE = aerobic exercise; W = week; M = minute; S = session; D = day; RPE = rating of perceived exertion; FIM = Functional Independence Measure; SAS = self-rating anxiety scale; SDS = self-rating depression scale; HADS-A = Hospital Anxiety and Depression Scale—Anxiety; PSSS = Perceived Social Support Scale; HADS-D = Hospital Anxiety and Depression Scale-Depression; PFT = Pulmonary Function Tests; FVC% = forced vital capacity; FEV1% = forced expiratory volume in 1 s; SAI = state anxiety questionnaire; PQSI = Pittsburgh Sleep Quality Index; SF-36 = Short Form-36; 6 MWT = six minute walk test.

**Table 2:** Summary of finding using GRADE quality assessment.

Outcome	Certainty Assessment							№ of Patients		Effect	Certainty
	№ of Studies	Study Design	Risk of Bias	Inconsistency	Indirectness	Imprecision	Other Considerations	Respiratory Rehabilitation	Standard Treatment	Absolute (95% CI)	
Physical function	4	RCT	serious ^b^	serious ^c^	not serious	serious ^a^	none	132	134	SMD 0.83. higher (0.58 higher to 1.09 higher)	⨁◯◯◯ Very low
Physical-health-related quality of life.	2	RCT	serious ^b^	serious ^d^	not serious	serious ^a^	none	95	96	SMD 0.02 higher (0.57 lower to 0.62 higher)	⨁◯◯◯ Very low
Mental-health-related quality of life	2	RCT	serious ^b^	serious ^c^	not serious	serious ^a^	none	95	96	SMD 0.06 lower (0.51 lower to 0.40 higher)	⨁◯◯◯ Very low
Depression	2	RCT	serious ^f^	serious ^d^	not serious	serious ^a^	none	49	49	SMD 0.16 SD higher (0.66 lower to 0.97 higher)	⨁◯◯◯ Very low
Anxiety	3	RCT	serious ^b^	not serious	not serious	serious ^a^	none	119	119	SMD 0.8 lower (1.23 lower to 0.37 lower)	⨁⨁◯◯ Low
Dyspnoea	2	RCT	not serious	not serious	not serious	serious ^a^	none	78	79	SMD 0.55 lower (0.87 lower to 0.23 lower)	⨁⨁⨁◯ Moderate
Physical activity intensity level	2	RCT	not serious	serious ^d^	not serious	serious ^a^	none	37	37	SMD 1.27 lower (2.23 lower to 0.32 lower)	⨁⨁◯◯ Low
Peripheral muscle performance of lower limp	3	RCT	not serious	not serious	not serious	serious ^a^	none	96	97	SMD 0.9 higher (0.6 higher to 1.2 higher)	⨁⨁⨁◯ Moderate
Pulmonary function	2	RCT	serious ^b^	very serious ^e^	not serious	serious ^a^	none	95	96	SMD 1.92 lower (5.8 lower to 1.97 higher)	⨁◯◯◯ Very low
Sleep quality	1	RCT	serious ^g^	not serious	not serious	serious ^a^	none	70	70	MD 0.5 lower (0.83 lower to 0.16 lower)	⨁⨁◯◯ Low
Social support	2	RCT	serious ^b^	serious ^e^	not serious	serious ^a^	none	49	49	SMD 0.79 higher (0.94 lower to 2.53 higher)	⨁◯◯◯ Very low

CI: confidence interval; MD: mean difference; SMD: standardized mean difference; RCT: randomized control trials; ^a:^ the included studies recorded a small sample size for both the control and intervention groups; ^b:^ participants were aware of all rehabilitation procedures; ^c:^ there is moderate heterogeneity in the involved studies; ^d:^ there is substantial heterogeneity in the study’s outcome; ^e:^ there is considerable heterogeneity in the studies; ^f:^ blinding was not feasible for participants and researchers in the study; only the evaluator who gave the link of questionnaires and data analyst were blinded for the treatment; ^g:^ information regarding the blinding of the participant and the assessor was not provided.

## Data Availability

Not applicable.

## Supplementary Materials

The following supporting information can be downloaded at: https://www.mdpi.com/article/10.3390/healthcare10112130/s1, Supplementary File S1: Search strategy and risk of bias assessment.
