# Supplementary material for: Effectiveness of Pulmonary Rehabilitation among COVID-19 Patients: A Systematic Review and Meta-Analysis
Source: Healthcare (Basel). 2022 Oct 26;10(11):2130. doi: 10.3390/healthcare10112130 (PMC9691193; doi:10.3390/healthcare10112130)
Supplement: Supplementary file 1 [file healthcare-10-02130-s001.zip › healthcare-1937370-supplementary.pdf]

Supplementary File S1

1. Search strategy

1.**PubMed**

(“physical activit*”[Title/Abstract] OR “exercise” [Title/Abstract] OR “rehabilitation” [Title/Abstract] OR “telerehabilitation” [Title/Abstract] OR “training” [Title/Abstract] OR “fitness” [Title/Abstract]) AND (“Covid-19” [Title/Abstract] OR “SARS-CoV-2” [Title/Abstract] OR “2019-nCoV” [Title/Abstract]).

2. **Scopus**

TITLE- ABS(“Physical activit*” OR “exercise” OR “rehabilitation” OR “telerehabilitation” OR “training”, “fitness”) AND TITLE- ABS(“Covid-19” OR “SARS-CoV-2” OR “2019-nCoV”).

3.ScienceDirect

(“Physical activity” OR “exercise” OR “rehabilitation” OR “telerehabilitation” OR “training”, “fitness”) (“Covid-19” OR “SARS-CoV-2” OR “2019-nCoV”).

4. Google Scholar

allintitle:(“Physical activity” OR “exercise” OR “rehabilitation” OR “telerehabilitation” OR “training” OR “fitness”) (“Covid-19” OR “SARS-CoV-2” OR “2019-nCoV”)

2 Research Question

Studies about the Effectiveness of respiratory rehabilitation among COVID -19 patients were selected based on the “PICOS” (PRISMA-P 2016) technique:

“PICOS”

P (population) = COVID-19 patients

I (Intervention) = respiratory rehabilitation

C (Comparison) = Standard treatment

O (Outcome) = physical function and quality of life

S (Study design) = Randomised controlled trial, and controlled clinical studies

3. Risk of bias

1. Xia 2021

Bias	Authors' judgement	Support for judgement
Random sequence generation (selection bias)	Low risk	"Permutated allocation sequences for 1:1 block randomisation (block size 10–14) stratified by the hospital were computer-generated by an independent statistician."
Allocation concealment (selection bias)	Low risk	"Allocation was concealed by central randomisation and only revealed after baseline assessment through a call to the study centre".
Blinding of participants and personnel (performance bias) All outcomes	High risk	"One patient in the control group had been randomised mistakenly because the assessor forgot to inform the allocator about the patient's ineligibility due to refusal to collaborate in baseline assessments."<br>"Patients and therapists were requested to not disclose allocation to assessors at any time during the study"
Blinding of outcome assessment (detection bias) All outcomes	Low risk	"Assessors left the study site after the baseline measurements. Allocators then contacted the study centre in the presence of the patient to reveal allocation". "Patients and therapists were requested to not disclose allocation to assessors at any time during the study".
Incomplete outcome data (attrition bias) All outcomes	Low risk	"Six patients of the intervention group (10%) did not complete the post-treatment assessment". "Two patients who discontinued the intervention, one because of chest pain and one for unspecified reasons, missed the post-treatment assessment but returned for the follow-up assessment". "contact had lost with four additional patients in the telerehabilitation programme group and five patients from the control group at the final follow-up". Intention to treat analysis was applied.
Selective reporting (reporting bias)	Low risk	Expected outcomes were reported
Other bias	Low risk	Other biases have not been identified

2. Kong 2020

Bias	Authors' judgement	Support for judgement
Random sequence generation (selection bias)	Unclear risk	"a single-centre, evaluator-blinded, randomized controlled trial". "Twenty-six eligible patients were consecutively and randomly assigned to the intervention group and the control group".
Allocation concealment (selection bias)	Unclear risk	"Twenty-six eligible patients were consecutively and randomly assigned to the intervention group and the control group"
Blinding of participants and personnel (performance bias) All outcomes	High risk	"blinding was not feasible for participants and researchers in the study; only the evaluator (who gave the link of questionnaires) and data analyst were blinded for the treatment".
Blinding of outcome assessment (detection bias) All outcomes	Low risk	The assessors were blinded.
Incomplete outcome data (attrition bias) All outcomes	Low risk	All participants completed the study
Selective reporting (reporting bias)	Low risk	Expected outcomes were reported
Other bias	Low risk	Other biases have not been identified

3. Liu 2020

Bias	Authors' judgement	Support for judgement
Random sequence generation (selection bias)	Low risk	"A randomized controlled trial". "Computer-generated allocation was applied".
Allocation concealment (selection bias)	Low risk	"The demographic characteristics of each subject were assessed before randomizing the subject. Odd numbers of patients were in the intervention group while even numbers of patients in the control group using a computer-generated allocation order"
Blinding of participants and personnel (performance bias) All outcomes	High risk	"Participants were aware of all rehabilitation procedures".
Blinding of outcome assessment (detection bias) All outcomes	Unclear risk	"Efforts have been made to blind assessors and participants to group allocation, however, the author states that this cannot be guaranteed".
Incomplete outcome data (attrition bias) All outcomes	Low risk	All participants completed the study
Selective reporting (reporting bias)	Low risk	Expected outcomes were reported
Other bias	Low risk	Other biases have not been identified

4. Liu 2021

Bias	Authors' judgement	Support for judgement
Random sequence generation (selection bias)	Unclear risk	Comment: "One hundred and forty qualified participants were picked at random, the participants were divided into two groups at random, a control group and a trial group, with an equal number of 70 patients in each group".
Allocation concealment (selection bias)	Unclear risk	Comment: "One hundred and forty qualified participants were picked at random, the participants were divided into two groups at random, a control group and a trial group, with an equal number of 70 patients in each group".
Blinding of participants and personnel (performance bias) All outcomes	Unclear risk	"Information regarding the blinding of the participant is not provided".
Blinding of outcome assessment (detection bias) All outcomes	Unclear risk	"The medical team for the trial group consisted of at least five qualified medical staff+.<br>Comment: the trial did not report if the assessor was blinded or not.
Incomplete outcome data (attrition bias) All outcomes	Low risk	All participants completed the study
Selective reporting (reporting bias)	Low risk	Expected outcomes were reported
Other bias	Low risk	Other biases have not been identified

5. Blanco 2021

Bias	Authors' judgement	Support for judgement
Random sequence generation (selection bias)	Low risk	"A randomized, controlled, parallel, double-blind, two-arm clinical trial of treatment. Patients were divided into two groups using balanced randomization, carried out with free software". "The principal investigator and auditor only performed the randomization sequence".
Allocation concealment (selection bias)	Low risk	"Patients were divided into two groups using balanced randomization, carried out with free software". "The principal investigator and auditor only performed the randomization sequence". "No participant in the study had access to the randomization sequence, which was hidden and saved, to guarantee correct randomization with security".
Blinding of participants and personnel (performance bias) All outcomes	Low risk	"Evaluators and patients in the study were blinded during the entire process". "The evaluator was unaware of the study objectives and the randomized distribution of patients to study groups, and he did not have access to the randomization sequence".
Blinding of outcome assessment (detection bias) All outcomes	Low risk	The assessors were blinded
Incomplete outcome data (attrition bias) All outcomes	Low risk	All participants completed the study
Selective reporting (reporting bias)	Low risk	Expected outcomes were reported
Other bias	Low risk	Other biases have not been identified

6. Gerez 2021

Bias	Authors' judgement	Support for judgement
Random sequence generation (selection bias)	Low risk	"A randomized, controlled, parallel, double-blind, two-arm clinical trial of the treatment. Patients were divided into two groups using balanced randomization". "The principal investigator and auditor only performed the randomization sequence".
Allocation concealment (selection bias)	Low risk	"Patients were divided into two groups using balanced randomization". "The principal investigator and auditor only performed the randomization sequence". "No participant in the study had access to the randomization sequence, which was hidden and saved, to guarantee correct randomization with security".
Blinding of participants and personnel (performance bias) All outcomes	Low risk	"The evaluators and patients in the study were blinded during the entire process". "The evaluator was unaware of the study objectives and the randomized distribution of patients to study groups, and he did not have access to the randomization sequence". "Meanwhile, although blinding for patients could not be achieved, subjects were unaware of the other treatment modalities". "They did not know if they belonged to the intervention or control groups".
Blinding of outcome assessment (detection bias) All outcomes	Low risk	The assessors were blinded
Incomplete outcome data (attrition bias) All outcomes	Low risk	All participants completed the study
Selective reporting (reporting bias)	Low risk	Expected outcomes were reported
Other bias	Low risk	Other biases have not been identified
